# Pediculosis in School Sitting: What Is the Role of School Nurses?

**Published:** 2017-09

**Authors:** Mohammed ALBASHTAWY

**Affiliations:** Community and Mental Health Department, Princess Salma Faculty of Nursing, AL al-Bayt University, Mafraq, Jordan

## Dear Editor-in-Chief

Pediculosis human’s capitis, more commonly known as head lice infestation, include adult lice, larvae (nymphs) and eggs that solely affect the human scalp (1–3). Pediculosis capitis is a widespread medical problem in both developing and developed countries and is considered as one of the community health concerns that affect school students, children, adolescences and adults (4, 5). Furthermore, the prevalence of infestation worldwide ranges from 5%–80% with the highest prevalence among preschool and elementary school children (2, 6).

Head lice infestation is found mainly in school settings and can cause itching, impetigo, and sensation of something moving in the hair; as well as irritability, sleeplessness and sores that are caused by recurrent scratching which, in turn, leads to secondary bacterial skin infection (2, 6). Moreover, this complication may lead to psychological distress for the infected children that affect their learning performance and academic achievements. Furthermore, absenteeism will increase because of perceived social stigma associated with head lice (1, 4, 5).

Pediculosis is considered as highly contagious, being spread mostly by direct physical contact between infected children. Spreading mostly occurs by sharing hair brushings, combs, sheets, hats, pillowcases, mattresses and clothes (4, 6). The pattern of spreading depends mainly on economic status, socio-demographic factors, hair characteristics, poor personal hygiene and over-crowding (2, 4, 6). Pediculosis control and treatment should be based on evidence-based methods because ignorance of proper treatment can be dangerous to the children and their families (4, 7). A lack of awareness in families, regarding head lice treatment, often leads to the use of traditional remedies including the direct use of insecticides or kerosene, which can be very harmful and may ultimately lead to the death of the individual being treated (4,6). American School Health Association (7) recommended that schoolchildren with live lice should remain in their classes but the school should discourage them from close direct physical contact with other schoolmates. Moreover, children with nits only should not be excluded from their classes (2, 7).

A school nurse is in a pivotal setting and position to serve in an advocacy role to the school students as a professional adviser and expert health care provider in nursing (5,7). The school nurse can follow up suitable measures and guidelines for serving assessment, planning, intervention and evaluation to tackle the problem of a head lice infestation (2,4). The school nurse can play a crucial role in providing health promotion and education programs in community or school settings, regarding the life cycle of head lice, methods of transmission, proper head lice identification and management, the complications associated with applying traditional remedies, and proper medical treatments (2,4,5). These programs can be conducted via workshops, lectures, posters, newsletters, conferences, and scientific days (7).

On the other hand, these programs can serve to prevent stigmatizing and dispel myths regarding pediculosis and reassure the infested children and their families that all treatment faces will be maintained under full privacy and confidentiality (5, 6). A school nurse must coordinate all the efforts with other health care providers, ministries of health and education, teachers, schools staff, and families regarding the health screening programs and campaigns (7–9). These programs and campaigns will be more effective if it is carried out early, and at the beginning of the academic year. Encouraging positive personal hygiene behaviors should be conducted by well-designed intervention programs, tailored to school children’ ages, preferences, to maximize their likelihood of engagement of such health promotion programs (8–9).
